# Exome sequencing analysis of gastric primary myeloid sarcoma with monocytic differentiation with altered immunophenotype after chemotherapy: case report

**DOI:** 10.1186/s13000-023-01311-1

**Published:** 2023-03-04

**Authors:** Xiang Li, Hongxia Zhang, Yong Cui, Haijun Zhang, Yonggang Wang, Meili Ding, Xingyao Zhu, Ruiqi Zhang, Qi Hu, Lin Tao, Wenhao Hu, Xinxia Li, A. O. Qilin, Hong Zou

**Affiliations:** 1 Department of Pathology, The First Affiliated Hospital, Shihezi University School of Medicine, Xinjiang, 832002 China; 2 Department of Hematology, First Affiliated Hospital, School of Medicine, Shihezi University, Shihezi City, 832008 Xinjiang Uygur Autonomous Region China; 3 Department of Pathology, Shihezi City People’s Hospital, Xinjiang, 832000 China; 4 Department of Pathology, Affiliated tumor Hospital of Xinjiang Medical University, Xinjiang, 830000 China; 5 Department of Pathology, School of Basic Medical Science, Institute of Pathology, Tongji Hospital; Tongji Medical College, Huazhong University of Science and Technology, Wuhan, 430030 Hubei China; 6 Department of Pathology, The Second Affiliated Hospital of Zhejiang University School of Medicine, Zhejiang, 310009 China

**Keywords:** Myeloid sarcoma with monocytic differentiation, Immunophenotype, Chemotherapy, Exome sequencing

## Abstract

**Background:**

Myeloid Sarcoma with monocytic differentiation is rare and quite likely is missed by surgical pathologists. However it is frequently misdiagnosed because of its non-specific imaging and histological pattern.

**Case presentation:**

We report the case of a 64-year-old woman with gastric primary myeloid sarcoma with monocytic differentiatio. Upper endoscopy revealed a neoplastic growth at the junction of the lesser curvature and gastric antrum. Except for a slightly increased peripheral monocyte count, no abnormalities were found on hematological and bone-marrow examination. Gastroscopic biopsy showed poorly differentiated atypical large cells with visible nucleoli and nuclear fission. Immunohistochemistry showed positive CD34, CD4, CD43, and CD56 expression, and weakly positive lysozyme expression. Immune markers for poorly differentiated adenocarcinoma, malignant melanoma, and lymphohematopoietic-system tumors were negative. The final diagnosis was myeloid sarcoma with monocytic differentiation. Chemotherapy did not shrink the tumor, so, radical surgery was performed. Although the tumor morphology did not change postoperatively, the immunophenotype did. CD68 and lysozyme expression (tumor tissue markers) changed from negative and weakly positive to strongly positive, AE1/3 expression (epithelial marker) changed from negative to positive, and CD34, CD4, CD43, and CD56 expression (common in naive hematopoietic cell-derived tumors) was greatly attenuated. Exome sequencing revealed missense mutations in *FLT3* and *PTPRB*, which are associated with myeloid sarcoma, and in *TP53*, *CD44*, *CD19*, *LTK*, *NOTCH2*, and *CNTN2*, which are associated with lymphohematopoietic tumors and poorly differentiated cancers.

**Conclusion:**

We diagnosed myeloid sarcoma with monocytic differentiation after excluding poorly differentiated adenocarcinoma, common lymphohematopoietic-system tumors, epithelioid sarcoma, and malignant melanoma. We identified that the immunophenotypic of patient had alterations after chemotherapy, and *FLT3* gene mutations. We hope that the above results will improve our understanding of this rare tumor.

**Supplementary Information:**

The online version contains supplementary material available at 10.1186/s13000-023-01311-1.

## Highlights

1. We used exome sequencing to detect and report the genetic changes in a case of extramedullary gastric primary myeloid sarcom with monocytic differentiationa.

2. Immunophenotypic alteration of tumor cells occurred after chemotherapy, suggesting that chemotherapy drugs induced tumor differentiation.

**Supplementary Information:**

The online version contains supplementary material available at 10.1186/s13000-023-01311-1.

## Background

Myeloid sarcoma most commonly consists of myeloblasts with or without features of promyelocytic or neutrophilic maturation, some of cases displays myelomonocytic or pure monoblastic morphology [1]. Myeloid sarcoma without acute myelocytic leukemia (AML) or hematological changes on bone marrow biopsy is defined as primary myeloid sarcoma, which is very rare. And primary myeloid sarcoma with monocytic differentiation involving extramedullary elements alone are even rarer. Its morphology is similar to that of undifferentiated carcinoma, non-Hodgkin lymphoma, and small round-cell sarcomas (e.g., neuroblastoma, rhabdomyosarcoma, Ewing sarcoma/primitive neuroectodermal tumor, and medulloblastoma) [2], and hence, it is commonly misdiagnosed. Here, we report a case of extramedullary myeloid sarcoma with monocytic differentiation in a non-leukemic patient. Although the upper gastrointestinal endoscopy and enhanced computed tomography (CT) findings were suggestive of malignancy, the tumor was diagnosed only after repeated biopsies, and histopathological and immunohistochemical examinations. The tumor failed to respond to chemotherapy, and was surgically removed. Interestingly, although the tumor morphology did not change postoperatively, its immunophenotype did, possibly because of chemotherapy-induced histiocytic differentiation of the naive tumor cells. Moreover, we are the first to report the molecular genetic changes of primary myeloid sarcoma with monocytic differentiation by using exome sequencing. Our patient had missense mutations in the *FLT3* and *PTPRB* genes, which are associated with myeloid sarcoma; missense mutations in *LTK* (associated with poorly differentiated cancer), *NOTCH2* (associated with diffuse large B-cell tumor), and *CNTN2* (associated with T-cell lymphoma); and a frameshift deletion mutation in the oncogene *TP53*. We also reviewed the relevant literature from 1990 to 2021, and identified 10 cases of myeloid sarcoma with monocytic differentiation. We have summarized their clinicopathological characteristics to improve our understanding of this disease.

## Case presentation

**Fig. 1:**
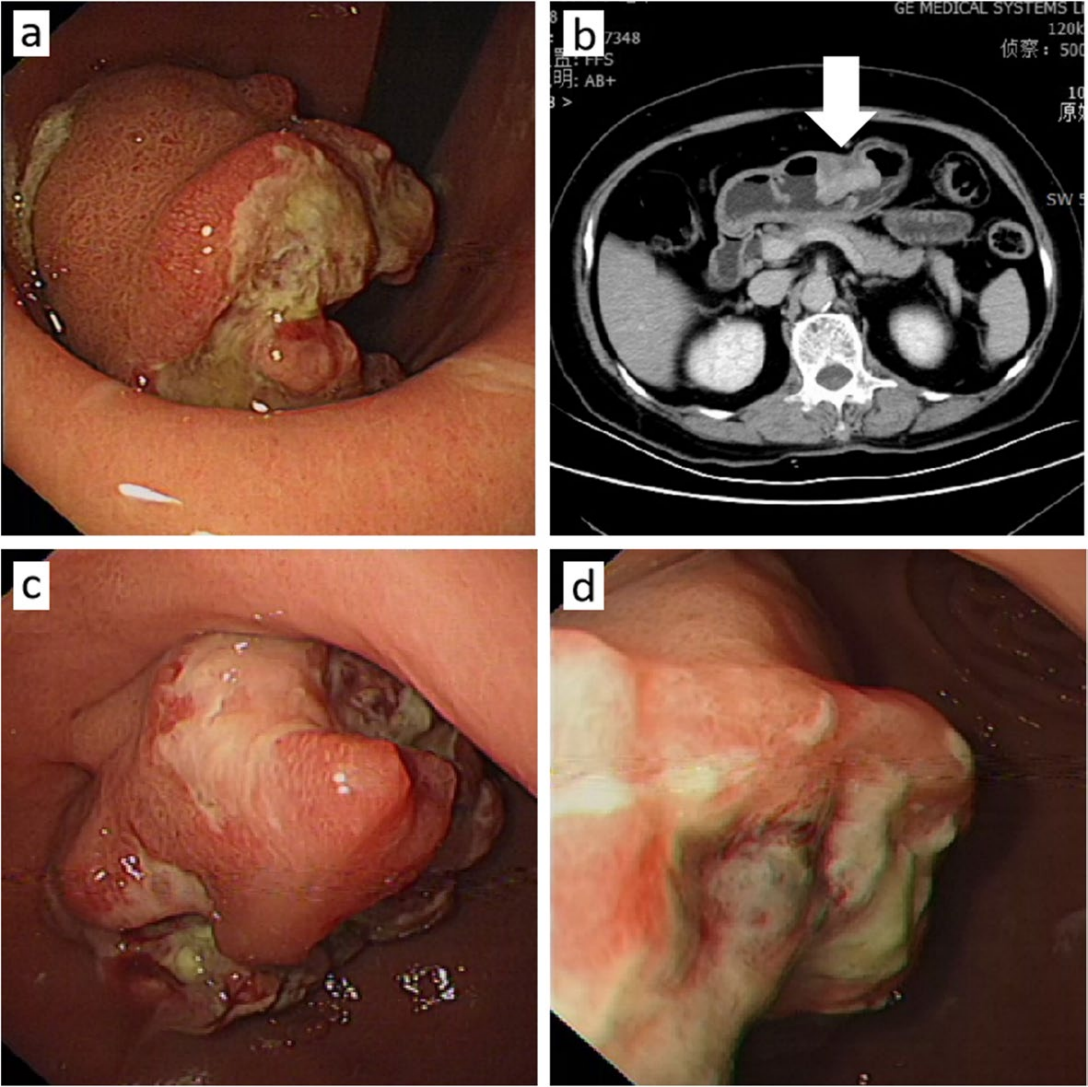
Endoscopy and computed tomography (CT) findings. **a** Endoscopy at the patient’s initial visit reveals a mass measuring 2.5 × 3.0 cm at the junction of the lesser curvature and gastric antrum. The mass has uneven surface mucosa and a base covered with a coating, with hard and brittle tissue that bleeds easily. **b** Enhanced CT of the upper abdomen shows limited thickening of the gastric wall in the gastric antrum and enlarged perigastric lymph nodes. **c** and **d** Endoscopy at 2 months after chemotherapy shows that the mass at the junction of the lesser curvature and gastric antrum is unchanged. It is of the same size (2.5 × 3.0 cm), and shows a central ulcer and a base covered with a coating, with hard and brittle tissue that bleeds easily

A 64-year-old woman presented with epigastric discomfort and erratic reflux since 2 months. Upper endoscopy revealed a 2.5 × 3.0 cm mass at the junction of the lesser curvature and gastric antrum (Fig. 1a). Computed tomography (CT) demonstrated an irregular soft-tissue masses projecting into the gastric lumen (Fig. 1b). Hematological examination showed elevated monocyte count, normal platelet count and globulin level.

**Fig. 2:**
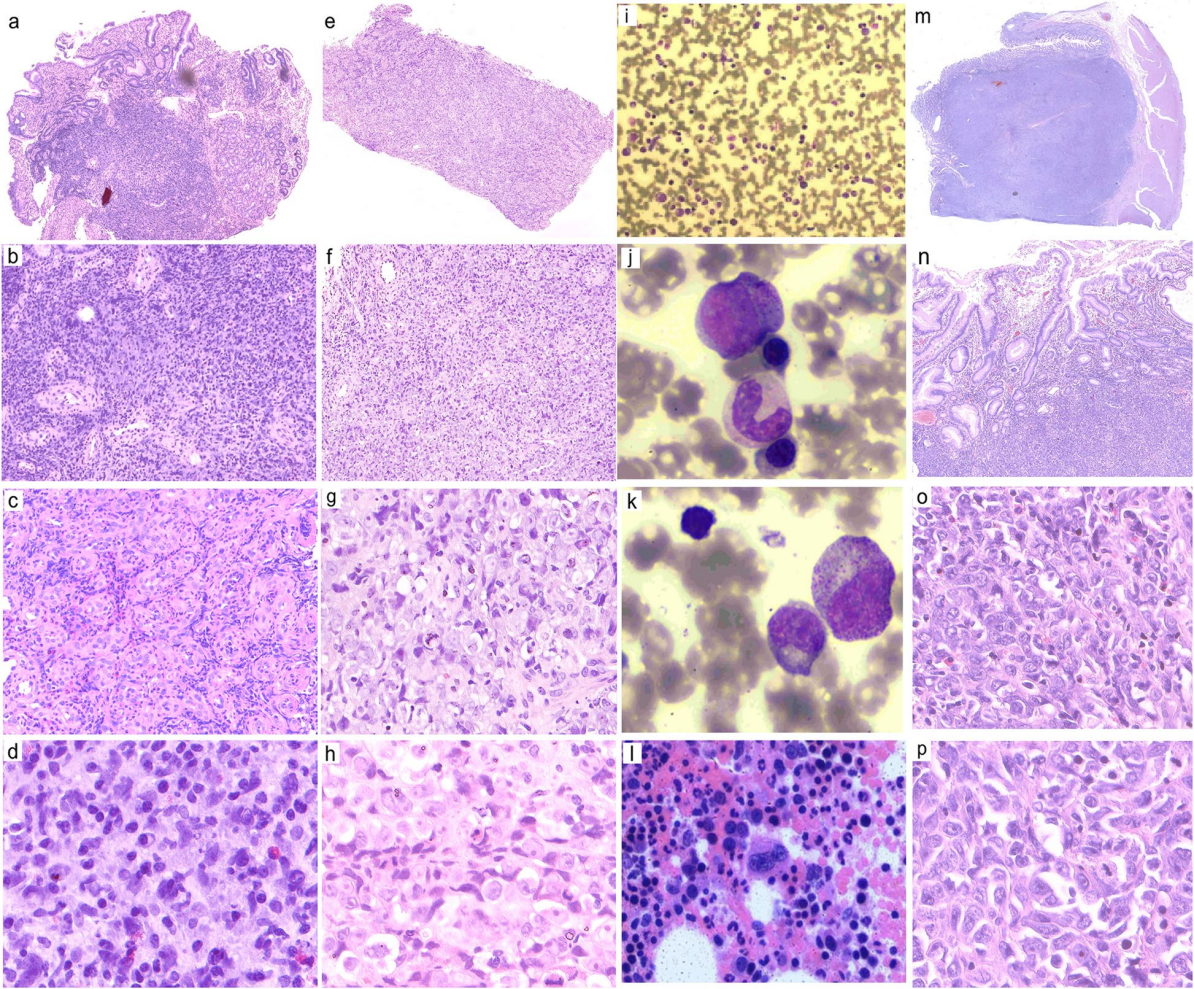
Microscopic findings. Submucosal infiltration of poorly differentiated large cells with flakes and cords. **a–h** Hematoxylin and eosin-stained images of preoperative tumor tissue (magnification: 2 × − 40×). Focal mucosal erosion with granulation-tissue hyperplasia and the submucosal infiltration of poorly differentiated atypical large cells are seen at low magnification. The tumor-cell cytoplasm appears slightly basophilic under high magnification, and some cells show obvious nucleoli and nucleolar division. Some tumor cells have large nuclei, and some nuclei are deviated, resembling plasma cells. Some tumor cells are vacuolated and resemble signet ring cells. Plasma cells and eosinophils infiltrate the area surrounding the tumor. **i–k** Bone-marrow smear shows active nucleated cell proliferation, active granulocyte proliferation, and active red-lineage proliferation. The lymphocyte, monocyte, and plasma-cell ratios and morphology are generally normal. No metastatic cancer cells are seen. **l** Bone-marrow histology shows no intact bone trabecular structures, active nucleated-cell proliferation, no metastatic tumor cells, active granulocyte proliferation, and no increase in primitive cells. Predominantly mature cells with a small amount of cytosolic enlargement are seen. Active proliferation of nucleated erythrocytes is observed, with scattered megakaryocytes, lymphocytes, and plasma cells. No fibrous tissue hyperplasia is seen. **m–p** Hematoxylin and eosin staining of postoperative tumor tissue (magnification: 2 × − 40×) shows a nested and lamellar distribution of tumor cells under the gastric mucosa. The tumor-cell morphology is similar to the preoperative morphology, with abundant, slightly basophilic cytoplasm, oval or slightly horseshoe-shaped nuclei, large red nucleoli, and nucleolar division

**Fig. 3:**
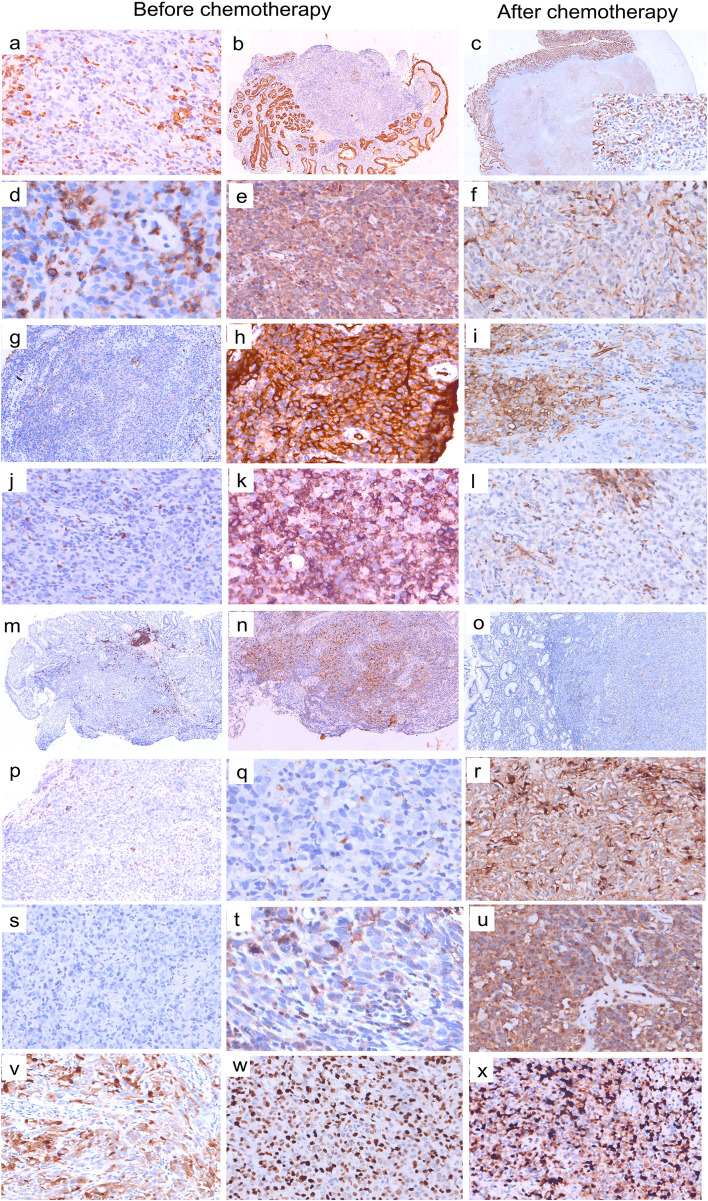
Comparison of preoperative and postoperative histopathology and immunohistochemistry. **a** Vimentin: negative (× 20); **b, c** AE1/3: shift from negative to positive (× 2); **d** LCA: negative (× 40); **e, f** CD4: from strongly positive to weakly positive (× 20); **g** MPO: negative (× 4); **h, i** CD34: from strongly positive to weakly positive (× 20); **j** CD3: negative (× 20); **k, l** CD43: from strongly positive to weakly positive (× 20); **m** CD20: negative (× 4); **n, o** CD56: from strongly positive to weakly positive (× 4); **p** CD38: negative (× 4); **q, r** CD68: from weakly positive to strongly positive (× 40; × 20); **s** CD117: negative (× 20); **t, u** Lysozyme: from weakly positive to strongly positive (× 40; × 20); **v** NSE: positive (× 20); **w** Ki-67: 70% (preoperative, × 20); **x** Ki-67: 80% (postoperative, × 20)

Endoscopic biopsy at low magnification showed the tumor cells poorly differentiated, atypical, large-cell lamellar and striated infiltrates. Under high magnification, tumor cells showed slightly basophilic cytoplasm, and some cells showed obvious nucleoli and nuclear fission. Some tumor cells had large nuclei, and some nuclei were deviated, resembling plasma cells (Fig. 2a–h). Some tumor cells were vacuolated and resembled signet-ring cells. This morphology was considered malignant and likely attributed to a poorly differentiated carcinoma or lymphoma. Most first-line markers were not expressed, such as AE1/3 (Fig. 3b), EMA, CK7, CAM5.2, and other epithelial-cell markers; CD34 (Fig. 3h) and CD56 were expressed, which excluded poorly differentiated adenocarcinoma. Among the lymphocytic, B-cell, and T/NK-cell markers, CD19, CD20 (Fig. 3m), CD79α, PAX-5, CD2, CD3 (Fig. 3j), CD5, and CD7 were negative, while CD56 was positive, which excluded diffuse large B-cell lymphoma and T/NK-cell lymphoma. The tumor cells were negative for CD38 (Fig. 3p), CD138, CD79α, MUM-1, BOB-1, and OCT-2, and showed no abnormality in the expression or ratio of kappa and lambda. The serum globulin count was normal, ruling out extramedullary plasmacytoma. MPO (Fig. 3g) was negative, excluding myeloid sarcoma with neutrophilic differentiation. Vimentin (Fig. 3a), CD117 (Fig. 3s), DOG-1, CD31, and ERG were negative, excluding epithelioid gastrointestinal stromal tumors and angiosarcomas. S-100, HMB45, and SOX-11 negativity excluded malignant melanoma and neurogenic tumors. The tumor cells were weakly positive for CD68 (Fig. 3q) and lysozyme, negative for CD163 (Fig. 3t), and positive for CD4 (Fig. 3e), which excluded rare tissue cell-derived tumors. No histocyte engulfment or skin lesions were observed. The platelet count was normal. CD1α, S-100, and CD123 negativity excluded blastic plasmacytoid dendritic-cell neoplasm, histiocytosarcoma, and Langerhans cell sarcoma. CD21 and CD35 were negative, excluding dendritic-cell sarcoma. Considering their CD34 expression, we speculated that the tumor cells originated from primitive or naive hematopoietic cells. CD43, a sensitive lymphatic marker, was positive (Fig. 3k). Further testing showed that the tumor cells were negative for TdT (excluding lymphoblastic lymphoma) and positive for NSE. Owing to the positive expression of NSE, CD4, CD56, and lysozyme, we considered a diagnosis of myeloid sarcoma with monocytic differentiation. Bone-marrow cytology and bone-marrow histology showed good hematopoiesis and no metastatic tumor cells (Fig. 2i-l). And the fusion genes commonly found in leukemia in bone-marrow tissue were all negative. So, we diagnosed the patient with primary extramedullary gastric myeloid Sarcoma with monocytic differentiation.

The patient began treatment with the DA regimen. After 2 months of treatment, repeat gastroscopy showed that the tumor mass was unchanged (Fig. 1c and d). The treatment was changed to decitabine combined with half-dose CAG. After 4 months of treatment, the tumor size remained unchanged on gastroscopy. Therefore, the patient underwent radical resection (distal major gastrectomy + Roux-en-Y anastomosis). The tumor surface was slightly cauliflower-shaped, the cut surface was grayish-white. Microscopy showed that the tumor had invaded the superficial muscular layer, and the gastric submucosa showed a nested and sheet-like distribution of tumor cells. The tumor-cell morphology was consistent with the preoperative biopsy findings, with abundant, slightly basophilic cytoplasm, oval or slightly horseshoe-shaped nuclei, large red nucleoli, and visible nuclear fission (Fig. 2m–p). Interestingly, immunohistochemical assays revealed a shift from negative to positive for AE1/3 and from weakly positive to strongly positive for CD68 and lysozyme. CD4, CD43, CD34, and CD56 positivity was greatly diminished (Fig. 3). Combined with the preoperative morphology and immunohistochemical results, we established a diagnosis of myeloid sarcoma with monocytic differentiation. The tumor cells showed mononuclear histiocytic differentiation, and this altered immunophenotype may be attributable to chemotherapy-induced tumor cell differentiation.

**Fig. 4:**
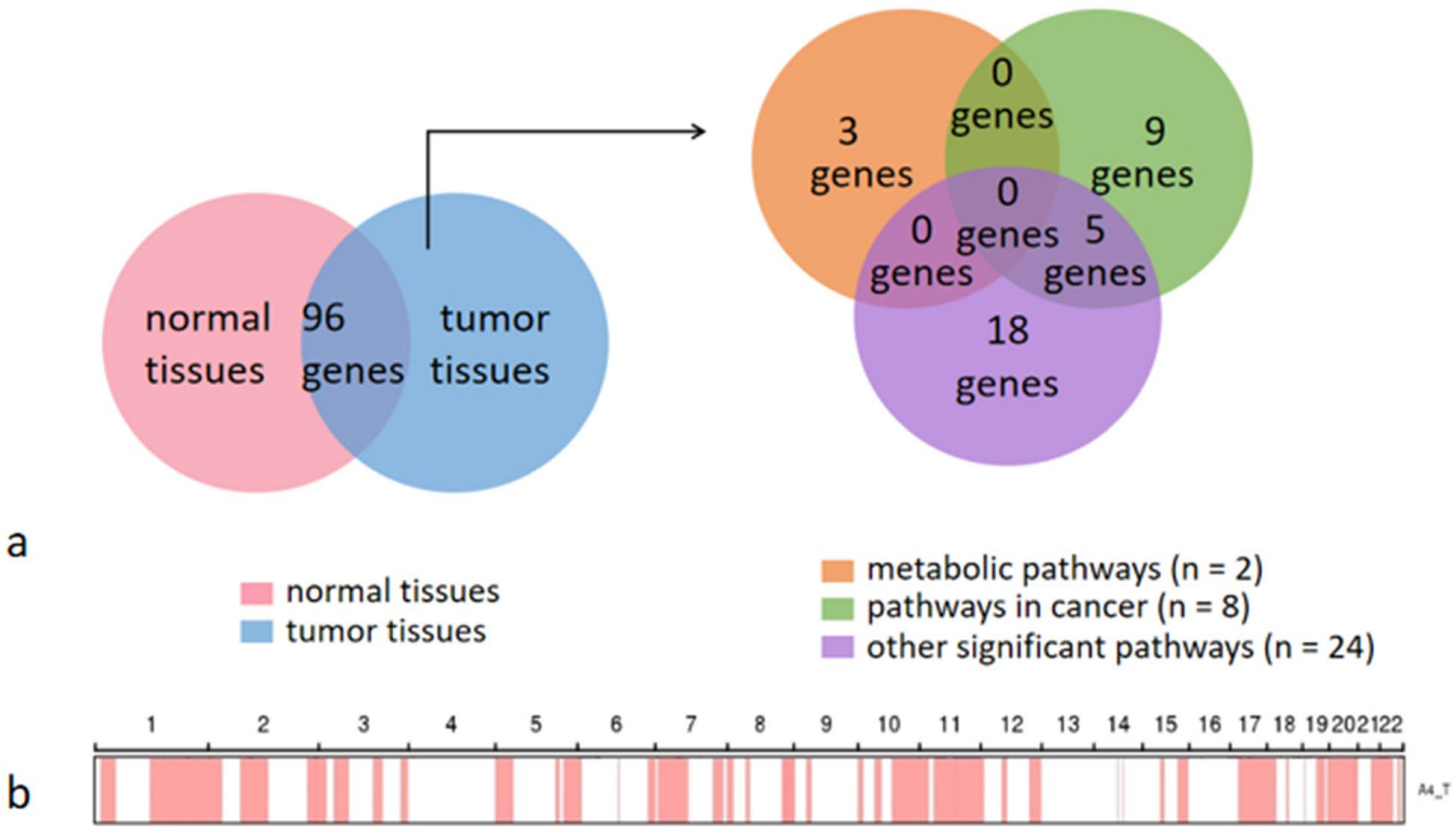
Venn diagram of differential genes. Among all genes with single nucleotide polymorphisms, we identified genes with missense mutations that were detected in tumor-tissue samples but not in normal-tissue samples

To determine the molecular genetic alterations in the tumor, we extracted DNA from the patient’s normal tissue and paraffin-embedded tumor tissue, performed exon sequencing, and analyzed germline mutations (SNPs and INDELs) using SAMtools (Table 1). In total, missense mutations SNPs and synonymous mutations SNPs were detected in the tumor tissue. Then screened for possible tumor-susceptibility genes: *MED23*, *PTPRB*, *ERG*, *PDE4DIP*, *FAT1*, *GRIN2A*, *CNOT1*, *WNK1*, *SH2B3*, *TJP2*, *MET*, *ANK3*, and *NKX3–1*. Annotation of the screening results revealed that the *PTPRB* gene (associated with angiosarcoma) had a missense mutation. Among genes with missense mutations, 96 differential genes were screened out. These genes were involved in 30 significant pathways according to KEGG pathway-enrichment analysis (Table 2). The tumor-associated pathway accounted for 33.3% genes (Fig. 4). The metabolism and other pathways accounted for 26.7 and 40% genes, respectively.

**Table 1:** The number of SNPs and different types of INDELs in different regions of the genome and in coding regions

*SNP*	*Sample*	*CDS*	*Synonymous* *SNP*	*Missense* *SNP*	*Stop gain*	*Stop loss*	*Unknown*	*Intronic*	*UTR3*	*UTR5*	*Splicing*	*ncRNA* *exonic*	*ncRNA intronic*	*ncRNA splicing*	*Upstream*	*Downstream*	*Intergenic*	*Others*	*Total*		
	N	20,884	10,848	9583	60	7	386	62,359	3487	1854	483	1872	3245	17	1396	611	8233	116	104,557		
	T	21,021	10,910	9663	64	8	376	66,146	3721	1963	495	1966	3537	18	1536	673	9518	122	110,716		
*INDEL*	*Sample*	*CDS*	*Frameshift* *deletion*	*Frameshift* *insertion*	*Non-frameshift* *deletion*	*Non-frameshift* *insertion*	*Stop gain*	*Stop loss*	*Unknown*	*Intronic*	*UTR3*	*UTR5*	*Splicing*	*ncRNA* *exonic*	*ncRNA* *intronic*	*ncRNA* *splicing*	*Upstream*	*Downstream*	*Intergenic*	*Others*	*Total*
	N	501	62	46	172	142	2	1	76	8844	512	226	118	167	395	4	204	82	915	13	11,981
	T	538	69	53	187	152	2	1	74	9388	530	240	122	176	432	3	238	88	1038	12	12,805

*SNP* Single nucleotide polymorphism, *INDEL* Insertion or deletion mutation, *ncRNA* Non-coding RNA

**Table 2:** Differential SNPs and major pathways between tumor tissues and normal tissues in our patient

*Type*	*Pathway*	*Gene name*	*P-value*
*Cancer pathways*	Pathways in cancer	AR,EGLN1,JAG2,NOTCH4,WNT9B	1.95 × 10^−5^
	Breast cancer	WNT9B,NOTCH4,JAG2	0.001
	Notch signaling pathway	JAG2,NOTCH4	0.001
	Signaling pathways regulating pluripotency of stem cells	WNT9B,LIFR	0.004
	Hippo signaling pathway	WNT9B,BMP6	0.004
	Wnt signaling pathway	NFATC4,WNT9B	0.005
	Jak-STAT signaling pathway	LIFR,CRLF2	0.005
	MAPK signaling pathway	RPS6KA4,MAPK8IP3	0.015
	Basal cell carcinoma	WNT9B	0.040
	Renal cell carcinoma	EGLN1	0.044
*Metabolic pathways*	Metabolic pathways	PDE10A,PDE8A,ATP6V0A1,DEGS2,B3GNT4,FPGS	0.001
	Purine metabolism	PDE8A,PDE10A	0.003
	Cushing syndrome	PDE8A,WNT9B	0.004
	Folate biosynthesis	FPGS	0.017
	Glycosphingolipid biosynthesis: lacto and neolacto series	B3GNT4	0.018
	Sphingolipid metabolism	DEGS2	0.030
	Ovarian steroidogenesis	BMP6	0.031
	Cortisol synthesis and secretion	PDE8A	0.041
*Others*	Cytokine-cytokine receptor interaction	BMP6,LIFR,CRLF2	0.001
	Human papillomavirus infection	WNT9B,ATP6V0A1,NOTCH4	0.001
	RNA degradation	C1D,MPHOSPH6	0.001
	Morphine addiction	PDE8A,PDE10A	0.002
	Endocrine resistance	JAG2,NOTCH4	0.002
	Collecting duct acid secretion	ATP6V0A1	0.018
	Circadian rhythm	B3GNT4	0.020
	Antifolate resistance	FPGS	0.020
	*Vibrio cholerae* infection	ATP6V0A1	0.032
	Epithelial cell signaling in *Helicobacter pylori* infection	ATP6V0A1	0.044
	Antigen processing and presentation	KLRC2	0.049
	Synaptic vesicle cycle	ATP6V0A1	0.049

We detected frameshift deletions and frameshift insertions of INDELs in the tumor tissue. CNVs were detected on chromosomes 1, 7, 8, 11, 16, 17, and 19 (Additional file 1: Table 1). The *MYC* gene, which is associated with cancer pathways, was located on chromosome 8. The oncogene *TP53* was located in the affected segment on chromosome 17. Finally, we compared somatic mutations with known driver genes in the database, and screened out the following possible driver genes in the tumor sample: *TP53*, WHSC1, and *SMARCA4* (Table 3). Further analysis of the sequencing results revealed missense mutations in the *FLT3* and *PTPRB* genes, which are associated with myeloid sarcoma. Moreover, missense mutations were detected in *CD44* and *CD19* (associated with acute myeloid leukemogenesis), *LTK* (associated with poorly differentiated adenocarcinoma), *NOTCH2* (associated with diffuse large B-cell tumorigenesis), and *CNTN2* (associated with T-cell lymphoma; Table 3).

**Table 3:** Main genes with mutations in our patient

*Gene Name*	*FLT3*	*PTPRB*	*LTK*	*NOTCH2*	*CNTN2*	*TP53*	*CD44*	*CD19*
*Mutation type*	Missense mutation	Missense mutation	Missense mutation	Missense mutation	Missense mutation	Frameshift deletion	Missense mutation	Missense mutation
*Related disease*	Myeloid sarcoma	Myeloid sarcoma	Poorly differentiated carcinoma	Diffuse large B-cell lymphoma	T-cell lymphoma	Multiple cancers	Acute myelocytic leukemia	Acute myelocytic leukemia

Whole-transcriptome sequencing of RNA extracted was performed to test for rearrangements in 555 genes related to tumorigenesis. The results were all negative.

## Discussion and conclusions

Myeloid sarcoma is formed by the infiltration of primitive or naive myeloid cells in organs and tissues other than the bone marrow. The tumor can occur at all ages, and may involve various organs and tissues. It can occur in isolation or secondary to primitive-cell transformation in AML, myeloproliferative neoplasms (MPNs), or myelodysplastic syndromes (MDSs) [3].

We retrieved 10 cases of myeloid sarcoma with monocytic differentiation from 8 articles. The clinicopathological characteristics of these cases are summarized in Table 4. The histopathology of tumer cell is similar to our patient. Tumor cells were positive for CD68, lysozyme, CD43, and negative for CD20, TdT, EMA, MPO. Only two patients underwent genetic testing. One of them had *FIP1L1*-*PDGFRA* rearrangement, and the other patient had an MLL gene rearrangement (11q23/MLL translocation; Table 4). Chemotherapy had a variable effect on the disease. We found that 2 of the 7 patients who had undergone chemotherapy relapsed, while the survival time of the untreated patients was very short. However, for our patient, chemotherapy showed no significant efficacy after 4 months, so surgical resection of the mass. No recurrence, metastasis, or hematological disease has been detected during follow-up to date.

**Table 4:** Clinical and pathological characteristics of 10 patients with primitive monocytic sarcoma

*Case*	*Age*	*Sex*	*Site*	*Primary/secondary or concurrent leukemia*	*Bone marrow*	*Immunohistochemistry*		*Treatment*	*Genetic testing*	*Recurrence* *(months)*	*Survival* *(months)*	*Reference*
						*Positive*	*Negative*					
1	21	F	Intralesional	Combined leukemia	Abnormal cells 41.43%	CD45, CD117, Lysozyme, CD68, CD43, CD99(Localized+)	Mpo, CD4, Kappa, Lambda, CD3, CD20, CD138, CK, EMA, synaptophysin, GFAP, Vimentin	Refused chemo.	NA	4	NA	[3]
2	28	M	Intralesional	Primary	Primary and immature monocytes accounted for 5.50%	CD45, CD43, CD117(±), CD68, Lysozyme, CD99(Localized+), CD4(±)	Mpo, CD34, CD3, CD2, CD23, CD1a, AACT, CK, synaptophysin	Chemo.	NA	15	NA	[3]
3	13	M	Left alveolar, gingival	Primary	Three lines hyperplasia	CD68(KP-1), Lysozyme, CD117, Ki-67(60%)	MPO, CD34, LCA, CD20, CD45RO, TdT, CD99, CD138, CD56, S-100, synaptophysin, myoglobin	Chemo. (DA regimen)	NA	NA	5	[4]
4	33	M	External acoustic meatus	Secondary to leukemia	Three lines are normal	CD68(KP-1), Lysozyme, MPO(−/+), CD34, Ki-67(50%)	CD117, LCA, CD20, CD45RO, TdT, CD99, CD138, CD56, S-100, synaptophysin, myoglobin	Chemo. (DA regimen)	NA	NA	NA	[4]
5	62	M	Right upper arm	NA	NA	Vim, LCA, CD43, CD68, CD34, S-100, CD99	CK, MPO, HMB45, Melan-A, EMA	NA	NA	NA	NA	[5]
6	46	M	Peritoneum	Leukemia	NA	CD68, CD56, CD45, CD4, NPM, LCA	AE1/3, EMA, calreticulin, CD117, synaptophysin, CGA, MPO, CD123, PS100	Chemo. (cytarabine and idarubicin)	NA	NA	NA	[6]
7	21	M	Bilateral cervical lymph nodes	NA	NA	CD11C, CD14, CD43, CD68R	NA	Radio.	FIP1L1- PDGFRA rearrangement	NA	NA	[7]
8	4 m	NA	Skin	NA	0.245 for primary monocytes and juvenile monocytes	S-100, CD68, CD4, CD56, CD123, CD163, Ki-67(40%)	CD1, CD21, MPO	NA	NA	NA	10	[8]
9	49	F	Uterus	Leukemia	NA	CD15, CD43, CD45, CD68, Lysozyme, Ki-67(70%)	CD3, CD5, CD10, CD20, CD34, CD79a, CD117, CK, TdT, MPO	Chemo. (cytarabine and idarubicin)	MLL gene rearrangement	NA	NA	[9]
10	2	M	Lower jaw	Primary	No abnormalities	CD45, CD68, Lysozyme	CD3, CD20, CD99, TdT, MPO, CD138	Chemo. (cytarabine and anthracyclines)	NA	NA	NA	[10]

*Chemo* Chemotherapy, *F* Female, *M* Male, *m* Month, *NA* Not applicable, *Radio* Radiotherapy

The pathological tumor characteristics in our patient were similar to those reported in the literature: poorly differentiated atypical cells, nucleoli, and nucleolar division. Interestingly, the initial gastroscopic biopsy specimen was negative for AE1/3, weakly positive for CD68 and lysozyme, and positive for CD4, CD43, CD34, and CD56. After 4 months of chemotherapy, immunohistochemical analysis of the surgically resected tumor tissue revealed that the expression of the tissue-differentiation markers CD68 and lysozyme changed from weakly positive to strongly positive, that of AE1/3 changed from negative to moderately positive, and the positivity of CD4, CD43, CD34, and CD56 was greatly diminished. This suggested that the tumor cells underwent some degree of differentiation, possibly because of the chemotherapeutic drugs. Bohl et al. [11] reported that the DNA-hypomethylating agent decitabine induces differentiation and apoptosis in primary leukemic cells. Our patient was treated with decitabine during the pre-chemotherapy phase, so we speculate that the tumor cells may have differentiated from primitive/naive hematopoietic tumor cells to histiocytes, which resulted in the observed immunophenotypic changes.

It is very difficult to diagnose extramedullary primary myeloid sarcoma in the absence of hematological disease in the patien. Its diagnosis requires a combination of clinical, pathological, morphological, and immunohistochemical findings. In our patient, the diagnosis was made after the exclusion of poorly differentiated adenocarcinoma, lymphoma, histiocytic sarcoma, and blast ic plasmacytoid dendritic-cell tumor. The differential diagnosis is shown in Table 5.

**Table 5:** Differential diagnosis

	*Clinical manifestations*	*CD19*	*CD20*	*CD22*	*CD79a*	*PAX-5*	*CD163*	*CD68*	*CD4*	*CD56*	*Lysozyme*	*TIA-1*	*CD45(LCA)*	*TdT*	*S-100*
*Diffuse large B-cell lymphoma*	NS	+	+	+	+	+	NA	NA	NA	NA	NA	NA	NA	NA	NA
*Histiocytic sarcoma*	Fever, weight loss, rash or hepatosplenomegaly, decreased eosinophil count.	–	–	–	NA	NA	+	+	+	+	Gorky District +	–	+	–	+
*Blastic plasmacytoid dendritic-cell tumor*	Skin lesions (nodules, plaques, or abrasions), thrombocytopenia	–	–	NA	–	NA	NA	+	+	+	NA	NA	+	+	+
*Primitive monocytic sarcoma*	NS	–	–	NA	–	–	–	+	+	+	+	Scattered +	–	–	–

*NA* Not applicable, *NS* Non-specific

The main treatments for myeloid sarcoma include surgical resection, chemotherapy, radiotherapy, and bone-marrow transplantation, with chemotherapy being the preferred choice. Chemotherapy regimens usually used for AML are effective in prolonging patient survival in myeloid sarcoma and reducing the risk of conversion of myeloid sarcoma to AML [4].

*NPM1* mutation, the most common mutation in myeloid sarcoma [12], was not detected in our patient. However, the *FLT3* gene showed missense mutations, which are also associated with myeloid sarcoma. In 2017, Mori et al. [13] reported a new FLT3/AXL inhibitor, gilteritinib, which has the ability to block mutant *FLT3* in AML cells and animal models, and may be a potential treatment option for AML patients with *FLT3* mutations. In 2020, Lin et al. [14] attempted to treat hematological malignancies with genetically modified chimeric antigen receptor (CAR) T-cell therapy. CAR T-cell therapy has been approved by the US Food and Drug Administration for the treatment of relapsed/refractory (r/r) diffuse large B-cell lymphoma, relapsed acute lymphocytic leukemia, and r/r mantle-cell lymphoma. In AML, CAR T-cell therapy has multiple therapeutic targets, such as CD33, CD123, CD44, and CD19. In our patient, both *CD44* and *CD19* showed missense mutations, indicating that CAR T-cell therapy may be a potential follow-up treatment.

In summary, extramedullary myeloid Sarcoma with monocytic differentiation is extremely rare and easily misdiagnosed. The differential diagnosis includes malignant tumors such as poorly differentiated adenocarcinoma, common lymphohematopoietic-system tumors, epithelioid sarcoma, and malignant melanoma, which must be ruled out before establishing the diagnosis. We have reported for the first time the overall molecular genetic alterations in this type of tumor. By means of exome sequencing, we identified that the patient had *FLT3* gene mutations, which were related to the occurrence of myeloid sarcoma. These mutations are a potential candidate for targeted therapy.

## Supplementary Information

**Additional file 1: Table 1.** Results of statistical analysis of copy number variations (CNVs).

## Data Availability

The datasets used and/or analyzed during the current study are available from the corresponding author on reasonable request.

## Abbreviations

AML: Acute myelocytic leukemia
WHO: World Health Organization
CT: Computed tomography
HE: Hematoxylin-eosin staining
AE1/3: Pan Cytokeratin
CK7: Cytokeratin 7
EMA: Epithelial membrane antigen
CD: 1a, 2, 3, 4, 5, 7, 20, 21, 31, 34, 35, 38, 43, 45RO, 56, 68, 79a, 117, 138, 163
Cluster of differentiation: 1a, 2, 3, 4, 5, 7, 20, 21, 31, 34, 35, 38, 43, 45RO, 56, 68, 79a, 117, 138, 163
TIA-1: T-cell intracellular antigen-1
MPO: Myeloperoxidase
BOB-1: B-cell oct-binding protein 1
LCA: Leukocyte common antigen
TdT: Terminal deoxynucleotide transferase
SMA: Smooth muscle actin
NSE: Neuron specific enolase
ALK: Anaplastic lymphoma kinase
