# Correction: Exome sequencing analysis of gastric primary myeloid sarcoma with monocytic differentiation with altered immunophenotype after chemotherapy: case report

**DOI:** 10.1186/s13000-023-01326-8

**Published:** 2023-03-28

**Authors:** Xiang Li, Hongxia Zhang, Yong Cui, Haijun Zhang, Yonggang Wang, Meili Ding, Xingyao Zhu, Ruiqi Zhang, Qi Hu, Lin Tao, Wenhao Hu, Xinxia Li, Qilin AO, Hong Zou

**Affiliations:** 1 Department of Pathology, The First Affiliated Hospital, Shihezi University School of Medicine, Xinjiang, 832002 China; 2 Department of Hematology, First Affiliated Hospital, School of Medicine, Shihezi University, Shihezi City, 832008 Xinjiang Uygur Autonomous Region China; 3 Department of Pathology, Shihezi City People’s Hospital, Xinjiang, 832000 China; 4 Department of Pathology, Affiliated Tumor Hospital of Xinjiang Medical University, Xinjiang, 830000 China; 5 Department of Pathology, School of Basic Medical Science, Institute of Pathology, Tongji Hospital; Tongji Medical College, Huazhong University of Science and Technology, Wuhan 430030, Hubei, China; 6 Department of Pathology, The Second Affiliated Hospital of Zhejiang University School of Medicine, Zhejiang, 310009 China

**Correction: Diagnostic Pathology 18, 35 (2023)**

**https://doi.org/10.1186/s13000-023-01311-1**

Following publication of the original article [1], the authors identified an error in the author name of Qilin AO. The given name and family name were erroneously transposed. The author also requested to replace Fig. 4.

The incorrect author name is: AO Qilin

The correct author name is: Qilin AO

The correct Fig. 4 is given below.

**Fig. 4:**
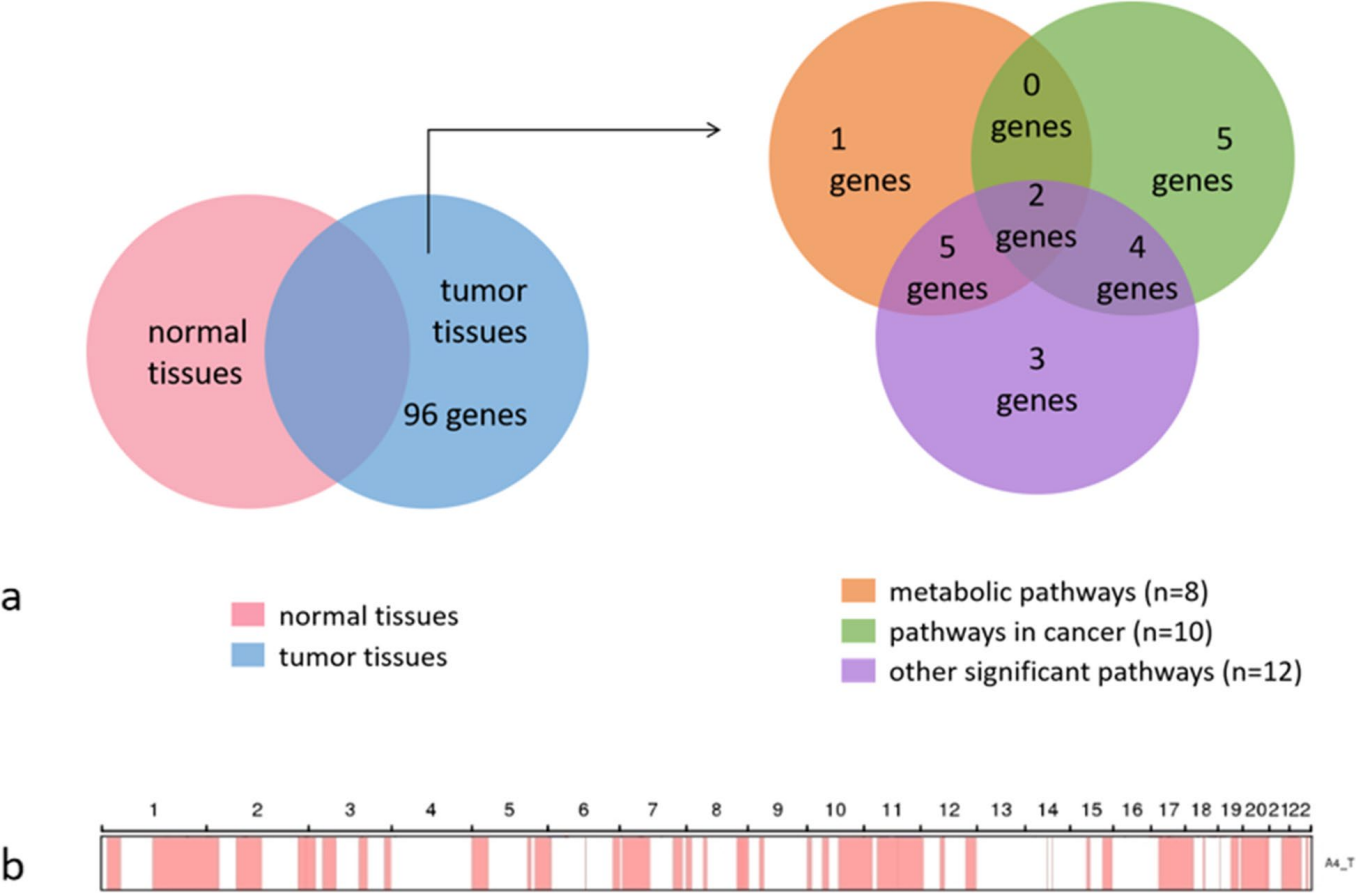
Venn diagram of differential genes. Among all genes with single nucleotide polymorphisms, we identified genes with missense mutations that were detected in tumor-tissue samples but not in normal-tissue samples

The original article [1] has been corrected.
